# Arabin cervical pessary for prevention of preterm birth in cases of twin-to-twin transfusion syndrome treated by fetoscopic LASER coagulation: the PECEP LASER randomised controlled trial

**DOI:** 10.1186/s12884-017-1435-0

**Published:** 2017-08-01

**Authors:** Carlota Rodó, Sílvia Arévalo, Liesbeth Lewi, Isabel Couck, Bettina Hollwitz, Kurt Hecher, Elena Carreras

**Affiliations:** 1 Maternal – Fetal Medicine Unit. Department of Obstetrics, Hospital Universitari Vall d’Hebron, Universitat Autònoma de Barcelona, Barcelona, Catalunya Spain; 2 Department of Obstetrics and Gynecology, University Hospitals Leuven, Leuven, Belgium; 3 Department of Development and Regeneration, KU Leuven, Leuven, Belgium; 4 Department of Obstetrics and Prenatal Medicine, University Medical Center Hamburg-Eppendorf (UKE), Hamburg, Germany

**Keywords:** Twin-to-twin transfusion syndrome, Fetoscopic LASER coagulation, Cervical length, Preterm delivery, Cervical pessary

## Abstract

**Background:**

Fetoscopic LASER coagulation of the placental anastomoses has changed the prognosis of twin-twin transfusion syndrome. However, the prematurity rate in this cohort remains very high. To date, strategies proposed to decrease the prematurity rate have shown inconclusive, if not unfavourable results.

**Methods:**

This is a randomised controlled trial to investigate whether a prophylactic cervical pessary will lower the incidence of preterm delivery in cases of twin-twin transfusion syndrome requiring fetoscopic LASER coagulation. Women eligible for the study will be randomised after surgery and allocated to either pessary or expectant management. The pessary will be left in place until 37 completed weeks or earlier if delivery occurs. The primary outcome is delivery before 32 completed weeks. Secondary outcomes are a composite of adverse neonatal outcome, fetal and neonatal death, maternal complications, preterm rupture of membranes and hospitalisation for threatened preterm labour. 352 women will be included in order to decrease the rate of preterm delivery before 32 weeks’ gestation from 40% to 26% with an alpha-error of 0.05 and 80% power.

**Discussion:**

The trial aims at clarifying whether the cervical pessary prolongs the pregnancy in cases of twin-twin transfusion syndrome regardless of cervical length at the time of fetoscopy.

**Trial registration:**

ClinicalTrials.gov Identifier: NCT01334489. Registered 04 December 2011.

## Background

Fetoscopic LASER coagulation of placental anastomoses (FLC) with or without Solomon technique [1] is the treatment of choice for severe Twin-to-Twin Transfusion Syndrome (TTTS) [2]. This procedure completely changed the natural history of TTTS [3]. However, although this first and most important step was taken and survival rates and neurological outcomes improved, premature births remain a serious concern: 41% of patients will deliver before 32 completed weeks [4]. Therefore, an effort to reduce prematurity is required. Cervical pessary placement could constitute an option to reduce prematurity, regardless of cervical length at the time of fetoscopy.

Transvaginal ultrasound permits the identification of twin pregnancies at high risk of preterm delivery [5, 6]. It is known that cervical length (CL) plays a role in the onset of preterm birth. The pathophysiology of premature ripening of the cervix varies between singleton and multiple pregnancies with twin pregnancies having a higher prevalence of short cervix at 23 weeks [7]. Regarding TTTS, Yamamoto et al. showed that short CL before treatment is an independent risk factor for preterm delivery [8].

The prematurity rate in cases of severe TTTS in patients with long cervix treated by FLC remains very high [9–11]. Although many cases are delivered prematurely for medical indications (mainly selective intrauterine growth restriction), a large proportion will also develop spontaneous preterm labour [12].

Information is lacking regarding the best therapeutic option in patients with short cervix who also require fetoscopy [9–11]. Salomon et al. suggested the use of an emergency cerclage in cases of TTTS with a short cervix at the time of surgery [13]. In this observational study, the emergency cerclage could prolong pregnancy and improve perinatal outcomes. It was the first study to demonstrate a potential benefit to prevent premature delivery in such cases. Nevertheless, emergency cerclage continues to be an invasive procedure requiring anaesthesia. It also bears the risk of infection, local inflammatory reactions and premature rupture of membranes, although these were not observed in the Salomon study [14–16]. In current practice an emergency cerclage is usually performed for a cervical length less or equal to 15 mm after fetoscopic surgery in cases of TTTS, although the benefit of the cerclage in these cases has been recently questioned [17].

Previous studies in singleton and twin pregnancies showed that the use of a cervical pessary significantly reduces the frequency of birth before 32 weeks and prolongs the pregnancy [18–20]. The advantages of using a cervical pessary are that it is a non-invasive, operator-independent, easy to use device, does not require anaesthesia and can easily be removed when necessary.

Our group performed a pilot study in which we placed an Arabin cervical pessary in cases of TTTS with FLC and retrospectively compared the outcomes with a control group. There was a lower rate of preterm birth and less neonatal morbidity in the pessary group [21].

The ProTWIN Trial [22], despite concluding that in unselected women with multiple pregnancies prophylactic use of a cervical pessary does not reduce poor perinatal outcome, found a significant difference in perinatal outcome for the subgroup of monochorionic pregnancies (14% vs. 25% in the control group).

The aim of this trial is to investigate whether a prophylactic cervical pessary will lower the incidence of preterm delivery in cases of twin-twin transfusion syndrome requiring fetoscopic laser coagulation.

## Methods/design

### Aims

We will perform a randomised trial (the PECEP LASER Trial: Arabin Cervical Pessary for prevention of preterm birth in cases of Twin-to-Twin Transfusion Syndrome treated by Fetoscopic Laser coagulation) to assess the effect of the Arabin cervical pessary on the rate of preterm delivery in cases of monochorionic pregnancies with twin-twin transfusion syndrome requiring fetal therapy.

This is a multicenter study to be conducted in the Hospital Universitari Vall d’Hebron in Barcelona (Catalunya, Spain), the Universitaire Ziekenhuizen in Leuven (Belgium), and the University Medical Centrer Hamburg-Eppendorf (UKE) in Hamburg (Germany). All centers have approval from the respective Medical Ethics Committees to conduct the trial.

The trial was registered in ClinicalTrials.gov in December 4th, 2011 (https://www.clinicaltrials.gov/ct2/show/NCT01334489?term=NCT01334489&recrs=a&rank=1). The trial identifier is NCT01334489.

The study is open for other centres who wish to participate.

### Participants

All women presenting with a monochorionic-diamniotic pregnancy with severe twin-twin transfusion syndrome between 16 and 26 weeks are eligible for the study with the exception of pregnancies complicated by major congenital abnormalities, uterine malformations, placenta praevia, active vaginal bleeding or spontaneous rupture of membranes at the time of randomization. Also patients with contraindication for pessary placement as painful regular uterine contractions, or a cervical cerclage in place will be excluded [20], as well as cases of demise of both twins after surgery.

Gestational age will be determined through menstrual history and first trimester scan.

### Procedures, recruitment, randomisation and collection of data

The Fetal Medicine Teams in the participating centres will counsel all women being referred for suspected TTTS. When fetoscopic LASER is planned, one of the Fetal Medicine specialists will confirm that the patient fulfils the inclusion criteria, and the study will be proposed. The patients will be informed about the intended therapeutic effect and possible side effects. The patient will be given an information sheet and if possible, she will have 24 h time to reflect on participation. If they agree, after obtaining their informed consent, they will be randomised in two groups: Usual management or cervical pessary placement.

The randomisation will be computed on-line at the platform for electronic data management based at the Catalan Institute of Pharmacology (ICF) where all data will be collected in a worldwide accessible online database. Each centre will have its own randomisation list and randomisation will be 1:1 for pessary placement versus control. Only a participant identification number in the electronic database will identify the participants. All documents will be stored securely and only accessible by trial staff and authorized personnel.

The study is not blinded.

Baseline characteristics of the women, the pregnancies and issues on the TTTS and the surgery will be recorded. The cervical length will be recorded before and after surgery and every 4 weeks until delivery. We will also record complications of the surgery, complications by the pessary and perinatal results.

### Intervention

For women allocated into cervical pessary group, the device will be inserted within 72 h after fetal surgery in the assessment room of the outpatients Department. This procedure does not require anaesthesia and does not need to be performed in an operating theatre.

All patients, both the pessary and the expectant management group, will be followed as in routine monochorionic follow-up, with clinical clinical review and ultrasound every two weeks. We will perform a transvaginal ultrasound for measuring cervical length every four weeks and will also assess the correct placement of the pessary.

Further surveillance of the pregnancy will not be influenced by the participation in the study. If the cervical length decreases later on during the pregnancy, the gestation will be managed according to the local protocol. However, cervical cerclage or progesterone will not be allowed in any case during the rest of the pregnancy.

The pessary will be removed at 37 weeks of gestation or before if any unexpected event or spontaneous delivery occurs. The indications to remove the pessary before this time will be: active bleeding, persistent contractions after tocolysis and premature rupture of the membranes after 34 weeks. After removing the pessary, the obstetrical management will be done as usual.

The study will close after the home discharge of the last newborn, as neonatal adverse outcomes will also be recorded.

### Outcomes

The primary outcome will be delivery before 32 completed weeks.

Secondary outcomes will be related to the delivery and perinatal results: preterm delivery before 28, 30, 34 and 37 weeks; preterm rupture of membranes; hospitalisation for threatened preterm labour (including the need of tocolysis and fetal lung maturation); fetal or neonatal death; composite neonatal morbidity (any of intraventricular haemorrhage, necrotising enterocolitis, retinopathy of prematurity, proven or suspected sepsis, need of Neonatal Intensive Care, need of ventilation, phototherapy, antibiotics or blood transfusion). We will also record significant maternal adverse effects, mainly due to the pessary.

A long-term follow-up of the babies is not planned.

### Statistics

#### Expected sample size

We based our sample size calculations on the assumption that the pessary reduces the primary outcome (preterm birth <32 weeks) from 40% in the control group to 26% in the pessary group [18]. With a study power of 80% and an alpha error of 5%, (assuming an effect size of 0.5 (moderate) and expecting a potential drop out rate of 10%), the required sample size has been estimated to be 352 patients, 176 cases in each arm of the study. We will perform 2 interim analyses, so the *p*-value would be set at 0.045 (O’Brien-Fleming rule) [23] instead of 0.05 and we would therefore need to recruit 182 patients in each arm.

#### Data analysis

A descriptive analysis by preterm birth will be carried out calculating means and medians for quantitative variables and proportions for categorical variables. T-test or Mann-Whitney test and Pearson Chi-squared or Fisher test will be used for comparing among exposure and outcomes groups per pregnancy. A multivariate logistic regression will be fitted to control for possible confounders. Relative risks and 95% confidence interval will be calculated for the outcomes.

For fetal and neonatal outcomes, multilevel models will be used to analyse continuous outcomes and generalized estimating equations will be used to assess categorical outcomes to adjust for the clustering of twins in mothers. A secondary analysis of length of gestation after fetoscopy will be plotted on a staggered-entry Kaplan-Meier curve where gestational age at birth (in days) will be the final event. Elective A Mantel-Cox test will be performed to test statistically for significant differences in the time interval between fetoscopy and birth. All analyses will be carried out with SPSS® 19.0 statistical package (IBM Company SPSS Inc. Headquarters, Chicago, Illinois. USA). Statistical significance will be accepted in all cases with a *p* < 0.05.

We do not expect to have lost data because this study has a short follow-up time. We will also perform a per-protocol analysis.

We also intend to perform a subgroup analysis according the cervical length: over 25 mm, 15 to 25 mm and less than 15 mm. We decided to perform the subgroup analysis with these cut-off lengths because 25 mm is around the 5th centile for cervical length for twins less than 22 weeks’ gestation [24] and some groups perform cervical cerclage in TTTS patients with CL below 15 mm [13].

#### Interim analysis

An interim analysis of efficacy and futility will be performed after each 100 patients enrolled in the study using the O’Brien & Fleming rule [23] for efficacy and conditional power calculation to assess futility. The trial will be stopped early if after the first 100 patients the difference has a *p*-value of <0.0001, if after 200 patients the *p*-value reaches a difference of <0.004. The interim analyses as well as the final outcome adjudication will be performed blinded to group assignment.

#### Study calendar

We expect patient recruitment to take approximately 3 years. One more year of duration is estimated for analysis and publication of the results.

## Discussion

Fetoscopic LASER coagulation of the placental anastomoses is the treatment of choice for severe twin-twin transfusion syndrome as this treatment has completely changed the prognosis in terms of survival of these babies. However, the morbidity of these babies is still very high, both at short and long term, mainly due to the high rates of prematurity.

The rate of spontaneous preterm delivery is high, even if the cervical length is in the normal range at the time of the surgery [25]. Almost half of these twins deliver around 32 weeks and most of them are electively delivered around 34 weeks [11, 12], thus we decided that the best cut-off should be “delivery before 32 weeks”, gaining at least two weeks in this group. Likewise, most of the babies are delivered preterm due to complications of the monochorionic placenta such as selective intrauterine growth restriction, twin anemia-policytemia sequence (TAPS) or recurrent TTTS. As this is an intention-to-treat study, the rate of iatrogenic births is expected to be the same in both groups.

In the last decade, some strategies to prevent preterm delivery in twins have been published: bed rest is not useful in twin pregnancies [26], and the use of the cervical cerclage has been questioned [27], despite some groups using it in cases of short cervical length after the fetal surgery [13].

The cervical pessary is a simple and easy-to-use method to prevent preterm birth that has been proven to be effective in singleton pregnancies [20], twins [18] and in the subgroup of monochorionic twins in the ProTwin Trial [22].

As far as we know, there is only one pilot study in this field [21], with favourable results for the pessary. Therefore, a trial that compares the use of the cervical pessary to expectant management in cases of twin-twin transfusion syndrome undergoing fetoscopic LASER coagulation is needed.

## Abbreviations

CL: Cervical length
EC: Ethics Committees
FLC: fetoscopic LASER coagulation
TAPS: twin anemia-policytemia sequence
TTTS: Twin-to-Twin Transfusion Syndrome
